# Acteoside and Isoacteoside Protect Amyloid β Peptide Induced Cytotoxicity, Cognitive Deficit and Neurochemical Disturbances In Vitro and In Vivo

**DOI:** 10.3390/ijms18040895

**Published:** 2017-04-24

**Authors:** Young-Ji Shiao, Muh-Hwan Su, Hang-Ching Lin, Chi-Rei Wu

**Affiliations:** 1 National Research Institute of Chinese Medicine, Ministry of Health and Welfare, Taipei 11490, Taiwan; yshiao@nricm.edu.tw; 2 School of Pharmacy, National Defense Medical Center, Taipei 11490, Taiwan; smh1027@syncorebio.com (M.-H.S.); lhc@sinphar.com.tw (H.-C.L.); 3 Sinphar Pharmaceutical Co., Ltd., Sinphar Group (Taiwan), Research & Development Center, I-Lan 26944, Taiwan; 4 Department of Chinese Pharmaceutical Sciences and Chinese Medicine Resources, College of Pharmacy, China Medical University, Taichung 40402, Taiwan

**Keywords:** acteoside, isoacetoside, amyloid β peptide, Morris water maze, acetylcholine, amyloid cascade

## Abstract

Acteoside and isoacteoside, two phenylethanoid glycosides, coexist in some plants. This study investigates the memory-improving and cytoprotective effects of acteoside and isoacteoside in amyloid β peptide 1-42 (Aβ 1-42)-infused rats and Aβ 1-42-treated SH-SY5Y cells. It further elucidates the role of amyloid cascade and central neuronal function in these effects. Acteoside and isoacteoside ameliorated cognitive deficits, decreased amyloid deposition, and reversed central cholinergic dysfunction that were caused by Aβ 1-42 in rats. Acteoside and isoacteoside further decreased extracellular Aβ 1-40 production and restored the cell viability that was decreased by Aβ 1-42 in SH-SY5Y cells. Acteoside and isoacteoside also promoted Aβ 1-40 degradation and inhibited Aβ 1-42 oligomerization in vitro. However, the memory-improving and cytoprotective effects of isoacteoside exceeded those of acteoside. Isoacteoside promoted exploratory behavior and restored cortical and hippocampal dopamine levels, but acteoside did not. We suggest that acteoside and isoacteoside ameliorated the cognitive dysfunction that was caused by Aβ 1-42 by blocking amyloid deposition via preventing amyloid oligomerization, and reversing central neuronal function via counteracting amyloid cytotoxicity.

## 1. Introduction

Alzheimer’s disease (AD), the most epidemic progressive neurodegenerative disorder, is characterized by behavioral disturbances such as cognitive deficits and neuropathological symptoms such as neuronal loss, senile plaques and neurofibrillary tangles [1]. Senile plaque contains fibrils that are compounds of amyloid β peptide (Aβ), which is formed from amyloid precursor protein (APP) via the amyloidogenic pathway [1,2]. When Aβ is oligomerized to amyloid fibrils and deposited in the brain especial entorhinal cortex and hippocampus, it causes cerebral neuronal loss, and particularly the degeneration of cholinergic neuronal circuits in the basal forebrain (BF) (cholinergic dysfunction) [3]. Therefore, recent researchers have suggested potential therapeutic approaches against AD that involve several disease-modifying strategies, such as blocking the cellular production of Aβ, preventing Aβ oligomerization, promoting Aβ degradation, and counteracting Aβ cytotoxicity [2].

**Figure 1:**
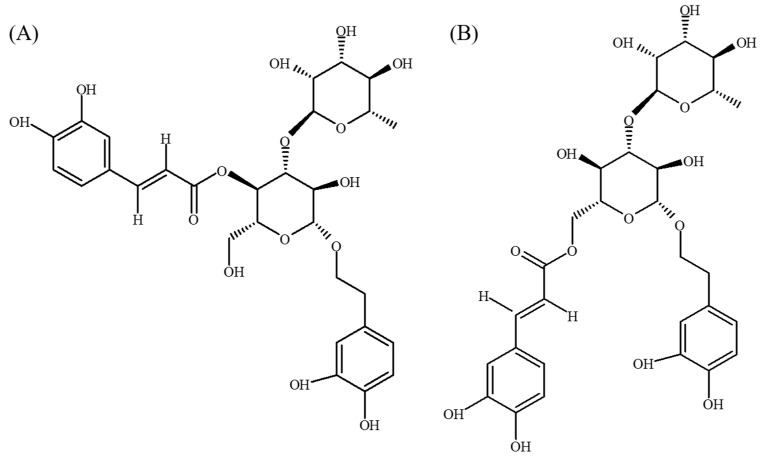
Structures of: (**A**) acteoside; and (**B**) isoacteoside.

Acteoside and its isomeric phenylethanoid glycoside, isoacteoside, (Figure 1) co-exist in various plants, such as *Cistanches* spp., *Castilleja* spp. and *Plantago* spp. [4,5]. Acteoside has been found to have antioxidative, anti-inflammatory, anti-nociceptive, anti-metastatic, hepatoprotective and cytoprotective activities [6,7,8,9,10,11,12]. Reports have shown that acteoside can alleviate acquired learning disability in mice that is induced by scopolamine [13], and reduce cerebral injury in mice that is induced by d-galactose [14,15]. Acteoside also shortens the escape latency in the Morris water maze (MWM) and reduces the number of retention errors in the step-down test in d-galactose plus AlCl_3_-induced mouse senescence model [16,17]. Acteoside protects neuronal damage caused by Aβ 25-35 in SH-SY5Y neuroblastoma cells [10,18] and inhibits the aggregation of Aβ 1-42 in vitro [19]. However, few studies have shown the effects of acteoside on Aβ 1-42-induced cognitive dysfunction in vivo and the pharmacological activities of isoacteoside. Therefore, this study investigates the effects of acteoside and isoacteoside on Aβ 1-42-induced behavioral changes following the osmotic intracisternal infusion of Aβ 1-42 into the lateral ventricle in rats. A meta-analysis of four behavioral tasks by Myhrer [20], acetylcholinergic and catecholaminergic activities strongly affect learning and memory. Aβ 1-42-infusion causes central acetylcholinergic and catecholaminergic dysfunction, which is closely related to memory deficits [21,22]. Therefore, this study further investigates the role of the central neurotransmitters in the acteoside- or isoacteoside-induced reversal of cognitive dysfunction that is caused by Aβ 1-42 infusion by measuring the levels of central neurotransmitters and the activities of related enzymes. Cognitive dysfunction and neurotransmitter disturbances in AD patients are closely associated with an amyloid cascade that involves amyloid generation, amyloid oligomerization and amyloid cytotoxicity [1,2,22]. The effects of acteoside and isoacteoside on amyloid generation, amyloid oligomerization and amyloid cytotoxicity are investigated in vitro to elucidate their memory-improving effects on Aβ 1-42-induced cognitive dysfunction.

## 2. Results

### 2.1. In Vivo Aβ 1-42-Infusion Model

#### 2.1.1. Effects of Acteoside and Isoacteoside on Behavioral Dysfunction Induced by Aβ 1-42 in Rats

**Figure 2:**
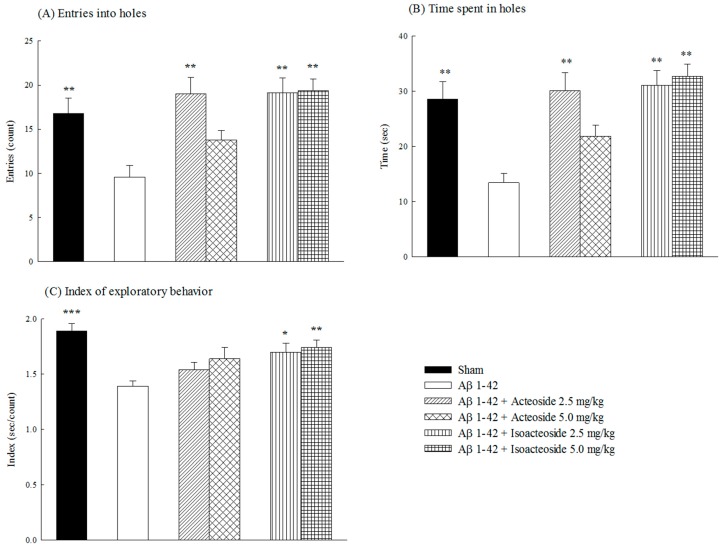
Effects of acteoside or isoacteoside (2.5, 5.0 mg/kg; po) on: (**A**) the number of entries into holes; and (**B**) time spent in holes; and (**C**) index of exploratory behavior in Aβ 1-42-infused rats. Exploratory test was performed on Day 7 following Aβ 1-42 infusion. Acteoside or isoacteoside was continuously administered after Aβ 1-42 infusion until all rats were sacrificed. Columns indicate mean ± SEM (*n* = 12). * *p* < 0.05, ** *p* < 0.01, *** *p* < 0.001 compared with Aβ 1-42-infused rats.

Aβ 1-42 infusion reduced the index of exploratory behavior, which incorporates time spent in the hole and the number of entries into the hole (*p* < 0.01, *p* < 0.001) (Figure 2A–C), but Aβ 1-42 infusion did not alter the movement time, distance or velocity of rats (Figure S1). Acteoside (2.5 mg/kg) or isoacteoside (2.5, 5.0 mg/kg) increased the time spent in the hole and the number of entry into the hole of Aβ 1-42-infused rats (*p* < 0.01) (Figure 2A,B), but only isoacteoside (2.5, 5.0 mg/kg) resorted the index of exploratory behavior (*p* < 0.05, *p* < 0.01) (Figure 2C). Neither acteoside nor isoacteoside at any dosage altered the motor activities of Aβ 1-42-infused rats (Figure S1).

**Figure 3:**
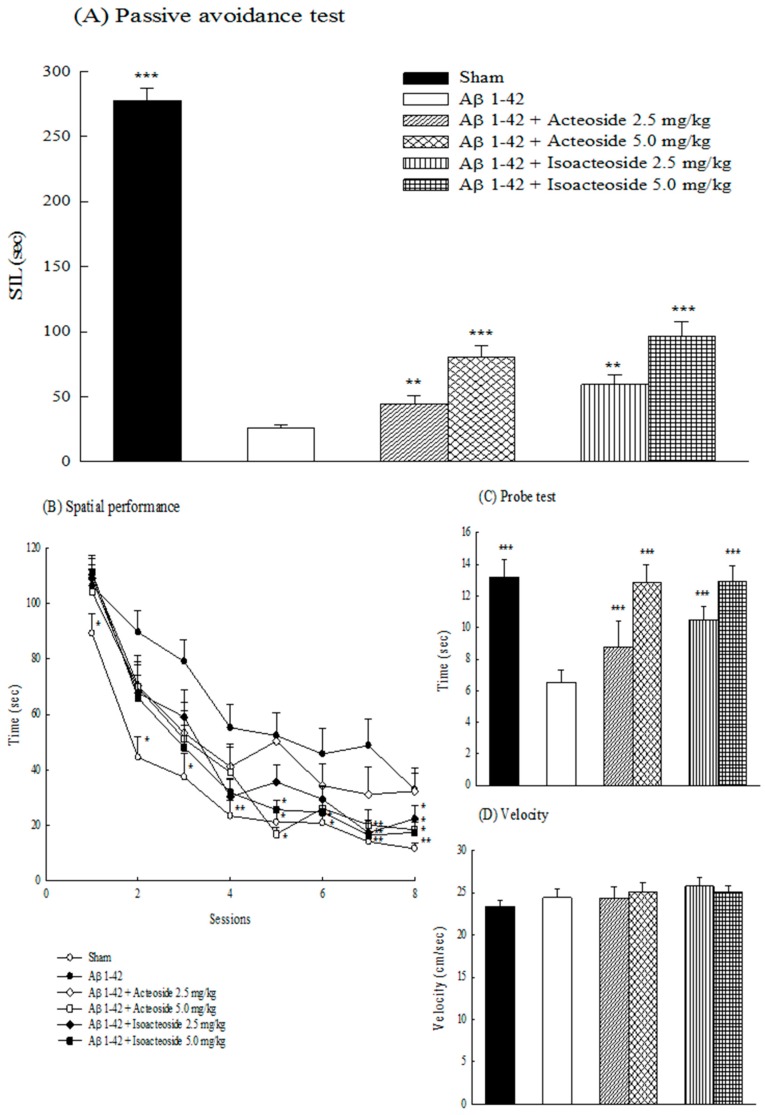
Effects of acteoside or isoacteoside (2.5, 5.0 mg/kg; po) on: (**A**) step-through latency (STL) of passive avoidance task; (**B**) spatial performance; (**C**) probe test; and (**D**) swimming velocity of MWM in Aβ 1-42-infused rats. Passive avoidance test was performed on Days 8–9 following Aβ 1-42 infusion. Spatial performance and probe test of MWM were performed on Days 10–14 following Aβ 1-42 infusion. Acteoside or isoacteoside was continuously administered after Aβ 1-42 infusion until all rats were sacrificed. Columns indicate mean ± SEM (*n* = 12). ** *p* < 0.01, *** *p* < 0.001 compared with Aβ 1-42-infused rats.

In a passive avoidance test, Aβ 1-42 shortened the latency of retention trial relative to the sham group (*p* < 0.001). Acteoside or isoacteoside (2.5, 5.0 mg/kg) prolonged the latency of retention trial in Aβ 1-42-infused rats (*p* < 0.01, *p* < 0.001) (Figure 3A). In MWM, the Aβ 1-42-infused group had a longer escape latency over eight trials on four training days (from Day 10 to Day 13 following Aβ 1-42 infusion) than the sham group (*p* < 0.05, *p* < 0.01). Aβ 1-42 infusion also shortened the time spent in the platform-quadrant from that of the sham group (*p* < 0.001) (Figure 3B,C). Both acteoside (5.0 mg/kg) and isoacteoside (2.5, 5.0 mg/kg) shortened the increase in escape latency that was caused by Aβ 1-42 infusion (*p* < 0.05, *p* < 0.01). Both acteoside and isoacteoside (2.5, 5.0 mg/kg) also prolonged the time spent in the platform-quadrant relative to Aβ 1-42-infused group (*p* < 0.001) (Figure 3B,C). However, the sham, Aβ 1-42-infused, acteoside- or isoacteoside-treated groups did not vary in swimming velocity (Figure 3D).

#### 2.1.2. Effects of Acteoside and Isoacteoside on Amyloid Deposition and Neurochemical Disturbances Induced by Aβ 1-42 in Rats

**Figure 4:**
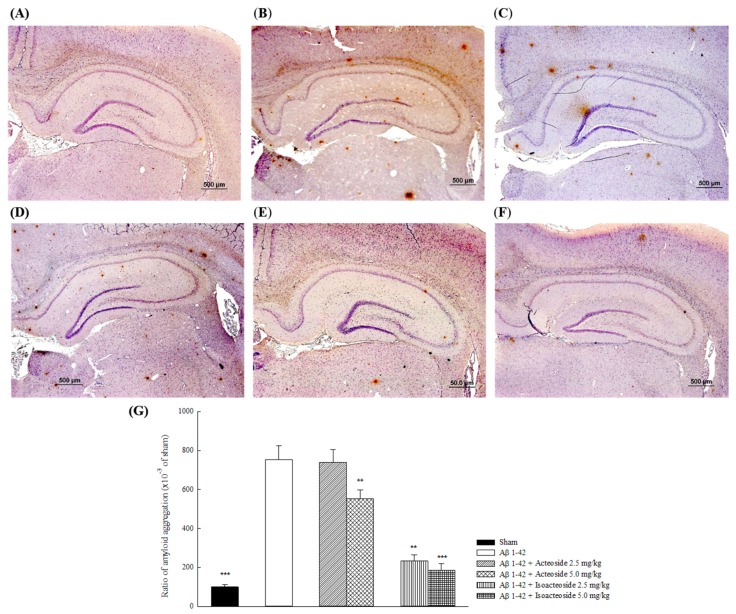
Effects of acteoside or isoacteoside (2.5, 5.0 mg/kg; po) on Aβ 1-42 deposition in Aβ 1-42-infused rats: (**A**) sham group; (**B**) Aβ 1-42-infused group; (**C**) acteoside (2.5 mg/kg)-treated group; (**D**) acteoside (5.0 mg/kg)-treated group; (**E**) isoacteoside (2.5 mg/kg)-treated group; and (**F**) isoacteoside (5.0 mg/kg)-treated group; and (**G**) ratio of amyloid deposition. Acteoside or isoacteoside was continuously administered after Aβ 1-42 infusion until all rats were sacrificed. Columns indicate mean ± SEM (*n* = 6). ** *p* < 0.01, *** *p* < 0.001 compared with Aβ 1-42-infused rats.

Figure 4 displays photographs of immunological staining and ratio of Aβ 1-42 deposition in the brain. The Aβ 1-42-infused group exhibited a significantly greater ratio of Aβ 1-42 deposition in the brain than the sham group (*p* < 0.01). Acteoside (5.0 mg/kg) or isoacteoside (2.5, 5.0 mg/kg) reduced the ratio of Aβ 1-42 deposition in the brain (*p* < 0.01, *p* < 0.001) (Figure 4G).

**Figure 5:**
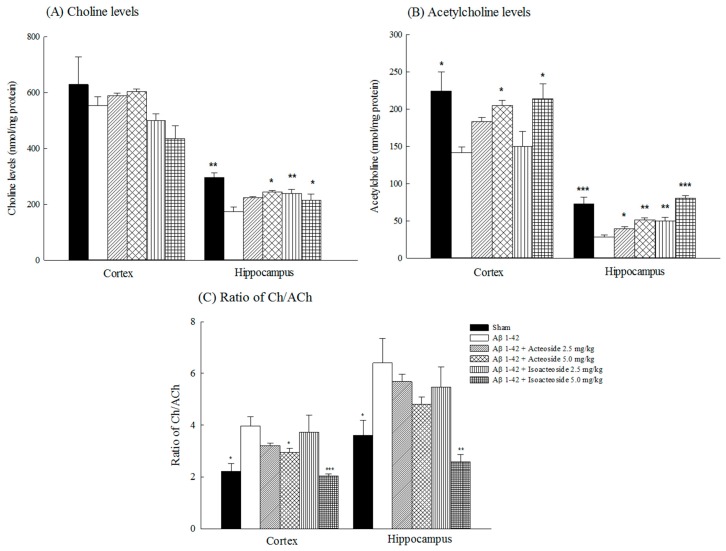
Effects of acteoside or isoacteoside (2.5, 5.0 mg/kg; po) on: (**A**) choline (Ch) levels; (**B**) acetylcholine (Ach) levels; and (**C**) ratio of Ch to Ach in cortex and hippocampus of Aβ 1-42-infused rats. Acteoside or isoacteoside was continuously administered after Aβ 1-42 infusion until all rats were sacrificed. Columns indicate mean ± SEM (*n* = 6). * *p* < 0.05, ** *p* < 0.01, *** *p* < 0.001 compared with Aβ 1-42-infused rats.

Aβ 1-42 infusion decreased the levels of cortical and hippocampal acetylcholine (Ach) (*p* < 0.05, *p* < 0.001) as well as hippocampal choline (Ch) (*p* < 0.01) (Figure 5A,B). Both acteoside and isoacteoside (2.5, 5.0 mg/kg) reversed the decline in hippocampal Ach levels that were caused by Aβ 1-42 infusion (*p* < 0.05, *p* < 0.01, *p* < 0.001), but only a dose of 5.0 mg/kg reversed the decrease in cortical Ach levels that was caused by Aβ 1-42 infusion (*p* < 0.05) (Figure 5B). Aβ 1-42 infusion reduced cortical and hippocampal dopamine (DA) levels (*p* < 0.01, *p* < 0.001), but only reduced hippocampal norepinephrine (NE) levels (*p* < 0.01) (Table 1). Only isoacteoside (2.5, 5.0 mg/kg) reversed the decline in hippocampal DA levels that was caused by Aβ 1-42 infusion (*p* < 0.001) (Table 1).

**Figure 6:**
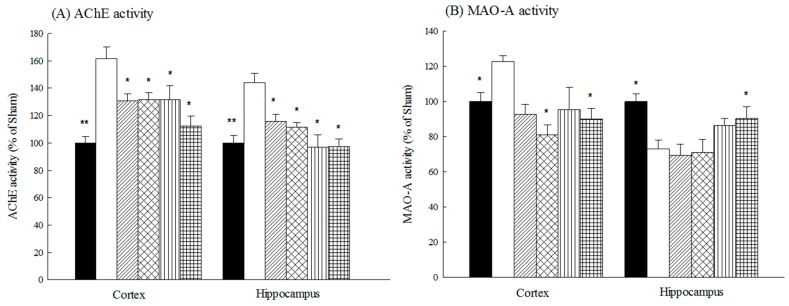

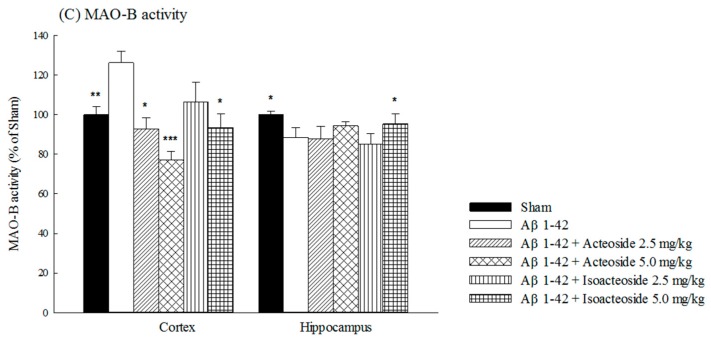
Effects of acteoside or isoacteoside (2.5, 5.0 mg/kg; po) on: (**A**) AChE; (**B**) MAO-A; and (**C**) MAO-B activities in cortex and hippocampus of Aβ 1-42-infused rats. Acteoside or isoacteoside was continuously administered after Aβ 1-42 infusion until all rats were sacrificed. Columns indicate mean ± SEM (*n* = 6). * *p* < 0.05, ** *p* < 0.01, *** *p* < 0.001 compared with Aβ 1-42-infused rats.

Aβ 1-42 infusion increased cortical and hippocampal acetylcholinesterase (AChE) activities (*p* < 0.05, *p* < 0.01). Both acteoside and isoacteoside (2.5, 5.0 mg/kg) prevented that increase in cortical and hippocampal AChE activity that would otherwise have been caused by Aβ 1-42 infusion (*p* < 0.05) (Figure 6A). Aβ 1-42 increased cortical monoamine oxidase-A (MAO-A) and MAO-B activities (*p* < 0.05, *p* < 0.01), but reduced hippocampal MAO-A and MAO-B activities in the rats (*p* < 0.05) (Figure 6B–C). Only isoacteoside at 5.0 mg/kg reversed the decrease in hippocampal MAO-A activity in Aβ 1-42-infused rats (*p* < 0.01) (Figure 6B,C).

### 2.2. In Vitro Test on Amyloid Cacade

#### 2.2.1. Effects of Acteoside and Isoacteoside on Neuronal Damage Induced by Aβ 1-42, and Intracellular and Extracellular Aβ 1-40 Levels in SH-SY5Y Cells

**Figure 7:**
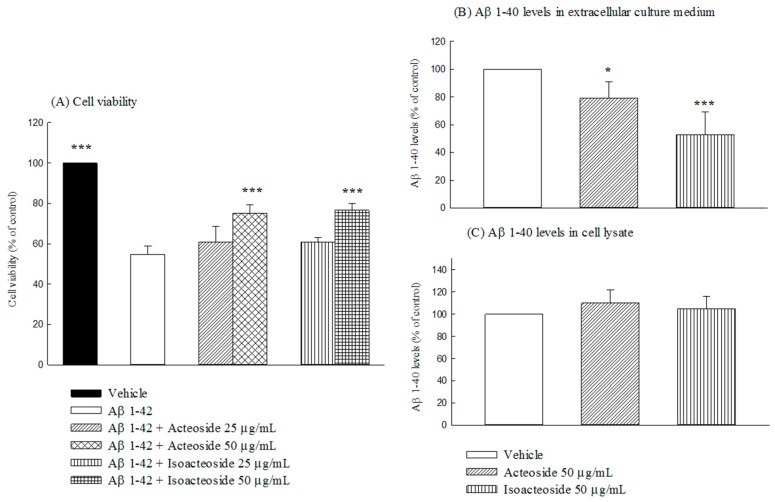
Effects of acteoside or isoacteoside (25, 50 μg/mL) on Aβ 1-42 toxicity, and extracellular and intracellular Aβ 1-40 levels in SH-SY5Y cells: (**A**) cell viability; (**B**) Aβ 1-40 levels in extracellular culture medium; and (**C**) Aβ 1-40 levels in cell lysate. Acteoside or isoacteoside was administered 1 h before treatment with Aβ 1-42. Columns indicate mean ± SD (*n* = 4). * *p* < 0.05, *** *p* < 0.001 compared with (**A**) Aβ 1-42-treated group or (**B**,**C**) vehicle group.

Incubation of SH-SY5Y cells with 20 μM Aβ 1-42 for 24 h reduced the cell viability to 52.73% of that of control cells (*p* < 0.001). Treatment with acteoside (50 μg/mL) or isoacteoside (50 μg/mL) recovered the cell viability that was reduced by Aβ 1-42 (20 μM) (*p* < 0.001) (Figure 7A). Both acteoside (50 μg/mL) and isoacteoside (50 μg/mL) reduced the extracellular Aβ 1-40 levels in SH-SY5Y cells (*p* < 0.05, *p* < 0.001), but did not alter the intracellular Aβ 1-40 levels (Figure 7B,C).

#### 2.2.2. Effects of Acteoside and Isoacteoside on Aβ 1-40 Degradation and Aβ 1-42 Oligomerization In Vitro

**Figure 8:**
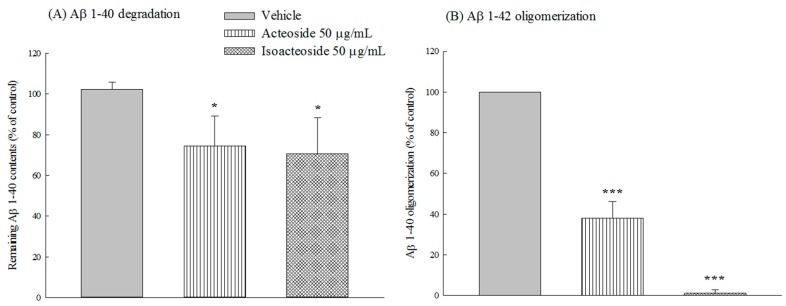
Effects of acteoside or isoacteoside (50 μg/mL) on: (**A**) Aβ 1-40 degradation; and (**B**) Aβ 1-42 oligomerization in vitro. Acteoside or isoacteoside was co-cultured with Aβ 1-40 or Aβ 1-42. Columns indicate mean ± SD (*n* = 4). * *p* < 0.05, *** *p* < 0.001 compared with vehicle group.

Both acteoside (50 μg/mL) and isoacteoside (50 μg/mL) increased the degradation of added synthetic Aβ 1-40 (10 ng) in SH-SY5Y-conditioned cell free medium in vitro (*p* < 0.05) (Figure 8A). Both acteoside (50 μg/mL) and isoacteoside (50 μg/mL) also reduced Aβ 1-42 oligomerization, as determined from the thioflavin T (ThT) binding fluorescence intensity in vitro (*p* < 0.001) (Figure 8B).

## 3. Discussion

Based on AD pathology, Aβ 1-42 is the critical protein in AD and intracisternal injection with Aβ 1-42 into rats produced memory impairment, morphological changes in the brain, and neuronal degeneration including cholinergic and monoaminergic systems [22,23]. The presented data reveal that intracisternal Aβ 1-42 infusion caused behavioral deficits including in the exploratory behavior, passive avoidance response, and spatial performance of MWM in rats. These results are consistent with our previous report and other reports [22,23,24]. Acteoside at a dose of 2.5–5.0 mg/kg ameliorated the deficits of passive avoidance learning and reference memory that were caused by Aβ 1-42, but only a dose of 5.0 mg/kg ameliorated the impairment of spatial performance. However, no dose of acteoside improved exploratory behavior. These memory-improving effects of acteoside are similar to those identified in other reports, which found that acteoside at 1.0–120 mg/kg reversed the memory impairment that was induced by scopolamine, d-galactose or d-galactose plus AlCl_3_ [13,14,16,17,25]. This difference between the results obtained herein with those other reports may be related to the given route and duration, and various models. Isoacteoside at 2.5–5.0 mg/kg exhibited a similar therapeutic potential against Aβ 1-42-induced behavioral dysfunction, but this effect of isoacteoside may differ from that of acteoside because isoacteoside reversed memory impairment partially by promoting exploratory behavior. Thus, we suggest that acteoside and isoacteoside may be potential anti-dementia phenylethanoid glycosides, and that these two stereoisomeric compounds exhibit similar memory-improving potentials but different behavioral-improving patterns against Aβ 1-42-induced behavioral dysfunction.

AD patients have complex neurochemical disturbances including of the catecholaminergic, cholinergic and glutaminergic neuronal systems [26]. AD patients have higher MAO-B activity than healthy controls, and this increased MAO-B activity may reflect abnormalities in the dopaminergic system [27]. In an AD-like animal model, Aβ 1-42 infusion into the lateral ventricle also caused memory deficits which were closely related to Aβ deposition and a subsequent cascade that caused, for example central cholinergic dysfunction in BF, including a decline in Ach levels and an up-regulation of AChE activity [22,28]. Thus, we further investigated the effects of acteoside and isoacteoside on Aβ-induced pathological changes, including amyloid deposition and neurochemical disturbances in rats. Our present data revealed similar pathological and neurochemical symptoms. Additionally, Aβ 1-42 infusion herein reduced cortical and hippocampal DA levels and hippocampal NE levels. The neurochemical changes were similar to those observed elsewhere [3,21,24]. Aβ 1-42 infusion was also found to cause a differential alteration of cortical and hippocampal MAO activities, mainly by elevating cortical MAO activities and reducing hippocampal MAO activities. Most related investigations have indicated that MAO-B activity was elevated around Aβ plaques (especially plaque-associated astrocytes), and have suggested the existence of a close positive correlation between MAO-B activity and amyloid plaques in the frontal cortex [27,29,30]. However, immunohistochemical studies have demonstrated MAO-B activities reflect disease-specific cellular changes in AD brain, and reduced MAO-B activities in advanced AD patients [31]. Researchers hole differing opinions regarding the alteration of MAO-A activities in AD patient. Recent reports have indicated that the alteration of MAO-A activities in AD patients may be related to presenilin-1 variants. Based on our results and others, we suggest that the alteration of regional MAO-A/B activities following Aβ 1-42 infusion may involve the regional activation of astrocytes around plaques sites and the loss of astrocytes/neurons with the progress of AD [27,31]. Aβ deposition causes up-regulates AChE activity around senile plaques, which favors the assembly of Aβ into fibrils, which cause Aβ cytotoxicity and, in particular, cholinergic and dopaminergic dysfunction [3]. Acteoside at 2.5–5.0 mg/kg reversed hippocampal Ach levels and inhibited the up-regulation of hippocampal AChE activity, but only at 5.0 mg/kg did it reduce Aβ deposition and reverse the disturbances of cortical Ach levels in Aβ 1-42-infused rats. Isoacteoside at 2.5–5.0 mg/kg reduced Aβ deposition and restored hippocampal cholinergic and dopaminergic neuronal function, including by blocking AChE up-regulating activity, but only at 5.0 mg/kg did it reverse the alteration of cortical and hippocampal MAO activities in Aβ 1-42-infused rats. Furthermore, acteoside at 5.0 mg/kg only restored the turnover rate of cortical Ach in Aβ 1-42-infused rats, whereas isoacteoside at 5.0 mg/kg restored the turnover rate of both cortical and hippocampal Ach (Figure 5C). From these above results, we suggest that the effects of acteoside and isoacteoside against Aβ 1-42-induced cognitive deficit may be related to reducing Aβ deposition, and then leading to a reversal of cortical cholinergic function, including an increase in the cortical Ach levels and a decrease in the Ach utility, by inhibiting AChE activity. Isoacteoside reduces Aβ deposition and Ach utility more than does acteoside. Unlike acteoside, isoacteoside restored cortical and hippocampal DA levels that were decreased by Aβ 1-42 infusion. Some researchers have pointed out that frontal and striatal DA levels are related to exploratory behavior [32,33,34]. Therefore, the memory-improving effects of isoacteoside may be further related to an improvement in exploratory behavior by the restoration of the dopaminergic function.

According to the amyloid cascade hypothesis, Aβ monomers are generated from APP via amyloidogenic pathway and secreted into the extracellular medium. Aβ monomers aggregated to form progressively larger species such as Aβ oligomers or fibrils under various physiological conditions, and are then deposited into senile plaques, causing neuronal dysfunction, such as neuronal apoptosis, and a decrease in long-term potentiation [2,3]. The results in this study reveal that acteoside at 50 μg/mL protected SH-SY5Y cells against Aβ 1-42-induced neural damage and inhibited Aβ 1-42 oligomerization, are revealed by ThT fluorescent staining. These results were consistent with earlier reports that acteoside protects Aβ 25-35-induced neural damage in SH-SY5Y and PC12 cells [10,18] and inhibits the fibril formation of Aβ 1-42 in vitro [19]. Acteoside was further found herein to reduce extracellular but not intracellular levels of Aβ 1-40, which was produced by amyloidogenic pathway in SH-SY5Y cells, and promoted Aβ 1-40 degradation in vitro. Hence, acteoside reduced extracellular Aβ 1-40 levels mainly by promoting Aβ 1-40 degradation. Isoacteoside possessed the same pharmacological potential to inhibit the amyloid cascade. The inhibiting by isoacteoside of amyloidogenesis and amyloid oligomerization exceeded that by acteoside. Some reports have indicated that acteoside has cytoprotective effects against Aβ 25-35, glutamate, okadaic acid, and MPP^+^ in vitro, and this effect may be mediated by their antioxidant and antiapoptotic activities by maintaining mitochondrial function and the activities of antioxidative enzymes, decreasing intracellular oxidative stress and Bax/Bcl-2 ratio, and inhibiting caspase-3 activity [9,10,18,35,36]. Other reports have indicated that acteoside protects Aβ 25-35-induced neuronal damage by inducting heme oxygenase-1 (HO-1) and the activation of transcription factor NF-E2-related factor 2 (Nrf2) by extracellular signal–regulated kinases (ERKs) and phosphatidylinositol 3-kinase/protein kinase B (PI3K/Akt) signaling [18], and restored the expression of neurotrophins including nerve growth factor (NGF), neurotrophin 3 (NT-3), and tropomyosin receptor kinase A (TrkA) in a d-galactose or d-galactose plus AlCl_3_-induced mouse senescence model [15,17]. NGF and NT-3 exhibited a neuroprotective function with therapeutic potential against neurodegenerative diseases [37]. NGF is synthesized by cortical and hippocampal neurons and retrogradely transported to BF cholinergic neurons through cholinergic projections that bearing the TrkA and low-affinity p75 neurotrophin receptor [38]. NGF maintains the survival of BF cholinergic neurons and enhances cholinergic neurotransmission through acute neurotransmitter-like and classical trophic mechanisms [39]. Accumulating evidence indicates that NGF improves the survival of cholinergic neurons and reduces cognitive decline in humans with mild AD [40]. Therefore, the linkage of NGF and ERK/Akt-Nrf2 signaling pathway on the memory-improving and cytoprotective effects of acteoside against Aβ 1-42 must be clarified and the cytoprotective mechanism of isoacteoside against Aβ-induced neural damage shall be investigated in the future.

## 4. Materials and Methods

### 4.1. Animals

Male Sprague-Dawley rats (300–350 g) were obtained from BioLASCO Taiwan Co., Ltd. They were housed in groups of four, chosen at random, in wire-mesh cages (39 cm × 26 cm × 21 cm) in a temperature (23 ± 1 °C) and humidity (60%) regulated environment with a 12 h–12 h light/dark cycle (light phase: 08:00 to 20:00). The Institutional Animal Care and Use Committee of China Medical University approved the experimental protocol (Protocol No. 99-127-B), and the animals were cared according to the Guiding Principles for the Care and Use of Laboratory Animals. After one week of acclimatization, the rats were used in the experiments that are described below.

### 4.2. Drugs

Acteoside and isoacteoside (with purities of greater than 98%) were kindly provided by Sinphar Pharmaceutical Co., Ltd. (I-Lan, Taiwan) and freshly dissolved in sterile distilled water. Synthesized human Aβ 1-42 and Aβ 1-40 were purchased from Tocris Bioscience (Ellisville, MO, USA). Aβ 1-42 was freshly dissolved with 35% acetonitrile/0.1% trifluoroacetic acid at a concentration of 250 pmol/μL and used to fill into mini-osmotic pump (Alzet 2002; Alza, Palo Alto, CA, USA) in vivo test. Aβ 1-42 and Aβ 1-40 were prepared with sterile phosphate buffer saline (PBS) in vitro test.

### 4.3. In Vivo Aβ 1-42-Infused Model

An Aβ 1-42-infused rat model was developed by infusing Aβ 1-42 into the cerebral ventricle via a mini-osmotic pump, as described elsewhere [23]. Briefly, rats were anesthetized with phenobarbital (45 mg/kg, i.p.) and placed in a David Kopf stereotaxic instrument. An infusion cannula was implanted into the left cerebral ventricle (AP-1.5, ML + 0.9, V-3.6 from Bregma), and a continual infusion of Aβ 1-42 (300 pmol/day) was maintained for at least two weeks by attaching an infusion cannula to the mini-osmotic pump. Sham group was infused with 35% acetonitrile/0.1% trifluoroacetic acid.

#### 4.3.1. Schedule of Aβ 1-42-Infused Model

Surgery, drug treatment, and behavioral tests were scheduled as in our previous report [24]. After implantation, Aβ 1-42 infusion began on a day that was designated as Day 0. On the next day (Day 1), the rats were orally administered with vehicle, acteoside or isoacteoside (2.5, 5.0 mg/kg) throughout Aβ 1-42 infusion period. The behavioral tests were carried out from Day 7 to Day 14 after Aβ 1-42 infusion, in the order, locomotor and exploratory tests (Day 7), passive avoidance test (Days 8–9), spatial performance test in MWM (Days 10–13), and probe test in MWM (Day 14). On Day 15 after Aβ 1-42 infusion, the rats were killed 1 h after their final treatment with acteoside or isoacteoside to measure AChE and MAO activities, levels of neurotransmitters and the metabolites in the brain.

#### 4.3.2. Behavioral Tests

The behavioral tests were performed as described in our previous report [23]. On Day 7, locomotor and exploratory tests were simultaneously performed with open-field task (Coulbourn Instruments L.L.C., Holliston, MA, USA). Each rat was observed for 10 min to record the movement time, distance and velocity (locomotor activity), the number of entries it made into the hole and the time spent (exploratory activity) using TruScan software v 2.07 (Coulbourn Instruments L.L.C.) [24]. On Day 8, the training trial of passive avoidance test was performed with passive avoidance apparatus (Coulbourn Instruments L.L.C.). When the rat entered the dark compartment from the light compartment, the door was closed and an inescapable foot shock (0.8 mA for 2 s) was delivered through the grid floor. On the following day (Day 9), the retention trial of passive avoidance test was conducted. The rat was again placed in the light compartment and the latency was recorded [24]. An upper cut-off time of 300 s was set. On Days 10–13, the spatial performance in MWM was tested using a black circular stainless pool (with a diameter of 165 cm and a height of 60 cm) that was filled with water at 23 ± 1 °C to a depth of 35 cm. Each rat underwent eight training sessions over four consecutive days to find the Plexiglass hidden platform (with a diameter of 10 cm) that was submerged 1.0 cm below the surface of the water. The swim path and escape latency to the platform of a white rat in the black pool were recorded using a video camera and an automated video tracking system device equipped with EthoVision XT software (Noldus Information Technology, Leesburg, VA, USA) [24]. On the following day (Day 14), the probe test was performed to measure the reference memory. The platform was removed and the parameters, including the time spent and distance moved in each quadrant while searching for the platform [24].

#### 4.3.3. Assessment of Aβ 1-42 Deposition, Neurotransmitter Levels, and AChE and MAO Activity in Brain

The rats in each group were separated into three groups: one for assaying Aβ 1-42 deposition, one for measuring neurotransmitter levels, one for measuring biochemical activities. To assay Aβ 1-42 deposition in the rat brain, the paraffin brain slices of rat were prepared and cut into sections (10 μm) using a microtome (Leica 2030 Biocut, Nussloch, Germany). The sections were labeled with a mouse anti-human amyloid β protein 17–24 monoclonal antibody (1:300, Dakopatts A/C; Glostrup, Denmark) and developed with 0.05% diaminobenzidine using a Vectastain kit (Vector Laboratories, Burlingame, CA, USA). The Aβ 1-42 labeled plaques at least 20 fields of each brain section were counted under 40× magnification using an image analyzer (Leica, Q500MC, Nussloch, Germany). The ratio of Aβ 1-42 deposition was obtained from Aβ 1-42 labeled plaques for each brain section. To measure the neurotransmitter levels, all rats were sacrificed and their brains were separated into cortex and hippocampus, which were placed on ice, according to the protocol of Glowinski and Iversen [41]. The supernatants of the brain tissues were prepared through homogenization, filtration and centrifugation, and then the neurotransmitter (and their metabolite) concentrations of brain supernatants were measured by high-performance liquid chromatography with electrochemical detection (EICOM HTEC-500, Kyoto, Japan). To measure brain AChE and MAO activities, all brains also were cut into cortex and hippocampus, and then the brain supernatants were prepared by homogenization and centrifugation. The brain supernatants and recombinant AChE enzyme were incubated with 5,5′-dithiobis(2-nitrobenzoic acid), and the absorbance at 412 nm was measured following the addition of acetylthiocholine. AChE activity was expressed as U AChE per mg protein. Brain homogenates were incubated with 5 U/mL horseradish peroxidase, 100 μM amplex red, and the substrate (5 mM serotonin for MAO-A or 5 mM benzylamine for MAO-B) at 25 °C for 60 min. The fluorescence intensity was measured, and MAO-A and MAO-B activities were expressed as percentage of the corresponding values for sham rats [24]. The protein content of brain supernatants was quantified using Bio-Rad protein assay kit.

### 4.4. In Vitro Test on Amyloid Cacade

Human SH-SY5Y neuroblastoma cells were cultured in DMEM that was supplemented with 10% fetal bovine serum, 100 units/mL penicillin and 100 μg/mL streptomycin in a water-saturated atmosphere with 5% CO_2_ at 37 °C. Experiments were performed 24 h after the cells were seeded in 96- or 24-well sterile clear-bottom plates. For cytoprotective and amyloidogenic-inhibiting tests, acteoside or isoacteoside (25, 50 μg/mL) was dissolved with culture medium. For amyloid degradation and oligomerization, acteoside or isoacteoside (50 μg/mL) was dissolved with sterile phosphate buffer saline.

#### 4.4.1. Assessment of Cytoprotective Effect in SH-SY5Y Cells

Acteoside or isoacteoside was treated 1 h before Aβ 1-42 (20 μM). The reduction of 3-[4,5-dimethylthiazol-2-yl]-2,5-diphenyl-tetrazolium bromide (MTT) to insoluble formazan was used to evaluate cell viability. Briefly, 24 h after exposure to Aβ 1-42 in 96-well plate, the medium was replaced and MTT (0.5 mg/mL) was added to each well. After incubation for 2 h at 37 °C, the cells were washed with PBS, and DMSO was added. The absorbance at 570 nm was measured using an ELISA reader. Cell viability was expressed as a percentage of corresponding value for untreated cells, which served as the control group (designated 100% viable). Each of four independent experiments was performed in triplicate.

#### 4.4.2. Assessment of Intracellular and Extracellular Aβ 1-40 Levels in SH-SY5Y Cells

Following treatment with acteoside or isoacteoside, culture media and SH-SY5Y cells were collected separately and the levels of Aβ 1-40 therein were determined using human Aβ 1-40 immunoassay kits (Invitrogen, Carlsbad, CA, USA). Experiments were performed according to the protocol of the manufacturer of the kits.

#### 4.4.3. Assessment of Cell-Free Aβ 1-40 Degradation In Vitro

The culture medium that contained the proteases to degrade Aβ was collected and used for the cell-free assay of Aβ degradation. Ten nanograms of Aβ 1-40 was added to 300 μL culture medium that contained acteoside or isoacteoside, and incubated at 37 °C for 24 h. The remaining Aβ was then quantified using human Aβ 1-40 immunoassay kits.

#### 4.4.4. Assessment of Aβ 1-42 Oligomerization In Vitro

Aβ 1-42 (100 μM) was dissolved in F-12 medium that contained acteoside or isoacteoside, and incubated at 4 °C for 24 h to accelerate Aβ oligomerization. The reaction solution was mixed with 5 μM ThT, and was then incubated for 30 min. The intensity of fluorescence at an emission wavelength of 485 nm was measured under excitation at a wavelength of 450 nm.

### 4.5. Statistical Analysis

The data of passive avoidance response were analyzed by performing a Kruskal-Wallis non-parametric one-way analysis of variance, followed by Dunn’s test. One-way analysis of variance (ANOVA) and then Scheff’s test were applied to data concerning spatial performance, probe test, the ratio of amyloid deposition, the activities of AChE and MAO, the levels of central neurotransmitters and their metabolites, and cell viability and amyloidogenic test. Significant differences in all statistical evaluations were calculated using SPSS software (version 22, IBM, Armonk, NY, USA) and *p*-values < 0.05 were considered significance.

## 5. Conclusions

Based on our results and those presented elsewhere [13,16,18,19,35], we suggest that acteoside and isoacteoside are potential therapeutic phenylethanoid glycosides for AD. The memory-improving mechanism of acteoside and isoacteoside involves reducing Aβ deposition and Aβ cytotoxicity by inhibiting Aβ oligomerization through the catechol moiety [19] and promoting Aβ degradation, and then reversing cortical cholinergic dysfunction, which includes the inhibition of AChE activity. Isoacteoside is more effective than acteoside with respect to amyloidogenesis and amyloid oligomerization, and it exhibits a different behavioral-improving pattern against Aβ 1-42-induced behavioral dysfunction. In the future, molecular docking studies of acteoside and isoacteoside against amyloid protein should be conducted and the interaction between phenylethanoid glycosides and amyloid protein must be assayed.

## Figures and Tables

**Table 1:** Effects of acteoside and isoacteoside (2.5, 5.0 mg/kg; po) on the levels of cortical and hippocampal neurotransmitters and their metabolites in Aβ 1-42-infused rats.

**The Levels of Cortical Neurotransmitters and Their Metabolites (ng/g Protein)**					
-	MHPG	NE	DOPAC	HVA	DA
Vehicle	26.05 ± 1.11 *	15.63 ± 0.41	17.13 ± 0.85 *	3.76 ± 0.78 **	3.83 ± 0.16 **
Aβ 1-42	22.87 ± 0.81	13.61 ± 1.20	11.97 ± 0.66	2.90 ± 0.23	2.18 ± 0.11
Acteoside					
2.5 mg/kg	22.40 ± 1.24	13.08 ± 1.06	12.91 ± 0.66	2.87 ± 0.11	2.26 ± 0.06
5.0 mg/kg	23.84 ± 1.94	12.91 ± 0.67	13.25 ± 1.07	2.90 ± 0.15	2.23 ± 0.15
Isoacteoside					
2.5 mg/kg	31.71 ± 3.21 *	12.60 ± 1.56	12.53 ± 3.33	2.91 ± 0.58	2.67 ± 0.43
5.0 mg/kg	34.41 ± 4.81 *	14.18 ± 1.01	13.80 ± 3.03	2.74 ± 0.69	3.84 ± 0.47 *
**The Levels of Hippocampal Neurotransmitters and Their Metabolites (ng/g Protein)**					
	MHPG	NE	DOPAC	HVA	DA
Vehicle	622.30 ± 17.58	64.49 ± 1.65 **	5.90 ± 0.39	3.60 ± 0.20	5.41 ± 0.35 ***
Aβ 1-42	620.97 ± 25.79	51.96 ± 1.54	5.12 ± 0.36	3.24 ± 0.16	0.97 ± 0.07
Acteoside					
2.5 mg/kg	641.15 ± 23.10	53.16 ± 2.29	5.22 ± 0.27	3.26 ± 0.12	1.07 ± 0.15
5.0 mg/kg	631.13 ± 36.81	53.53 ± 1.83	5.32 ± 0.14	3.34 ± 0.10	1.17 ± 0.13
Isoacteoside					
2.5 mg/kg	689.99 ± 79.95	54.01 ± 6.20	4.98 ± 1.15	3.68 ± 0.46	5.28 ± 0.83 ***
5.0 mg/kg	574.02 ± 54.16	45.13 ± 3.36	4.13 ± 0.45	3.29 ± 0.25	5.66 ± 0.36 ***

Acteoside or isoacteoside was continuously administered after Aβ 1-42 infusion until all rats were sacrificed. Columns indicate mean ± SEM (*n* = 6). * *p* < 0.05, ** *p* < 0.01, *** *p* < 0.001 compared with Aβ 1-42-infused rats.

## Supplementary Materials

Supplementary materials can be found at www.mdpi.com/1422-0067/18/4/895/s1.

## Abbreviations

Aβ	Amyloid β peptide
Ach	Acetylcholine
AChE	Acetylcholinesterase
AD	Alzheimer’s disease
APP	Amyloid precursor protein
BF	Basal forebrain
Ch	Choline
DA	Dopamine
MAO	Monoamine oxidase
MTT	3-[4,5-dimethylthiazol-2-yl]-2,5-diphenyl-tetrazolium bromide
MWM	Morris water maze
NE	Norepinephrine
PBS	Phosphate buffer saline
ThT	Thioflavin T
