# Supplementary material for: Acteoside and Isoacteoside Protect Amyloid β Peptide Induced Cytotoxicity, Cognitive Deficit and Neurochemical Disturbances In Vitro and In Vivo
Source: Int J Mol Sci. 2017 Apr 24;18(4):895. doi: 10.3390/ijms18040895 (PMC5412474; doi:10.3390/ijms18040895)
Supplement: Supplementary file 1 [file ijms-18-00895-s001.pdf]

Supplementary Materials: Acteoside and isoacteoside protect amyloid β peptide induced cytotoxicity, cognitive deficit and neurochemical disturbances *in vitro* and *in vivo*

Young-Ji Shiao, Muh-Hwan Su, Hang-Ching Lin and Chi-Rei Wu

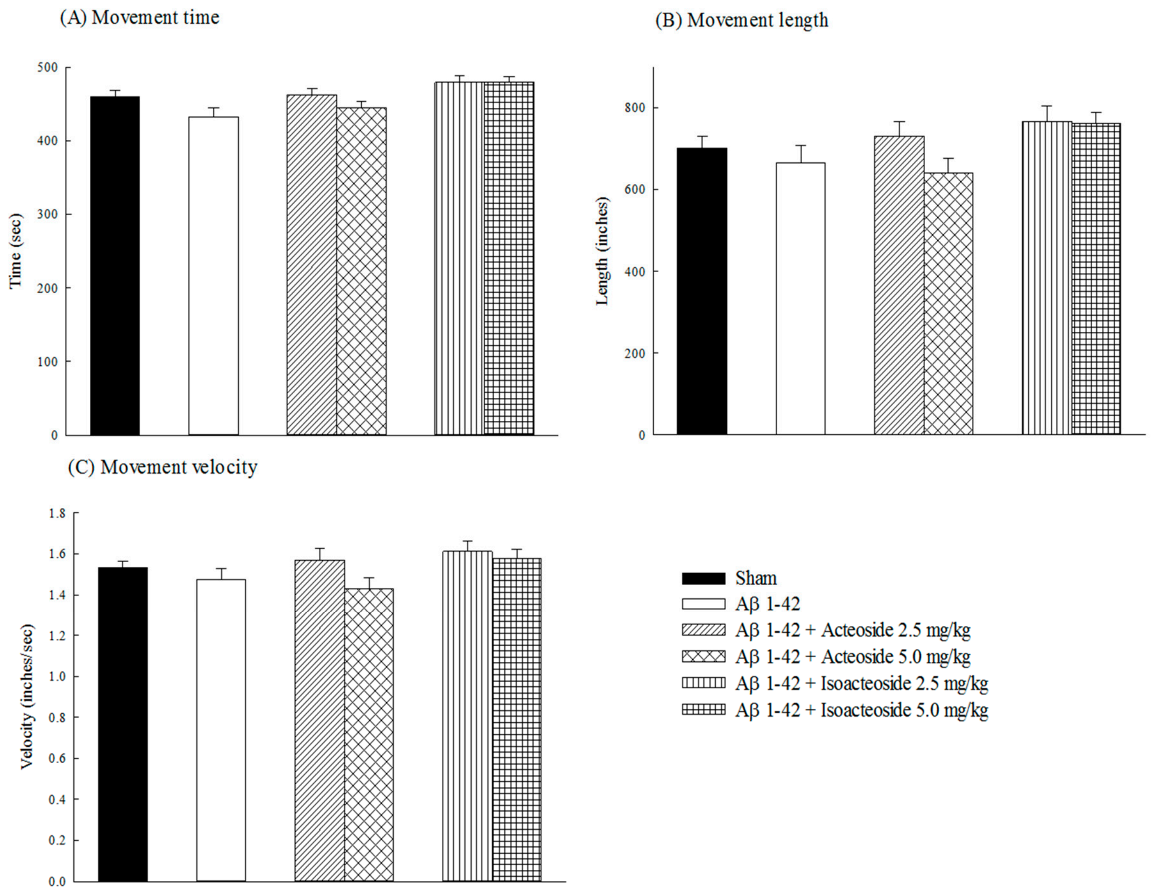

Figure S1. Effects of acteoside or isoacteoside (2.5, 5 mg/kg) on locomotor activity including (**A**) movement time, (**B**) movement length and (**C**) mean velocity, in Aβ 1-42-infused rats. Locomotor activity was performed on day 7 following Aβ 1-42 infusion. Acteoside or isoacteoside was continuously administered after Aβ 1-42 infusion until all rats were sacrificed. Columns indicate mean ± SEM (n = 6).
